# Human Pericardial Fluid-Derived Cells Exhibit Mesothelial-like Properties and Exert Proangiogenic Effects on Endothelial Cells

**DOI:** 10.3390/cells14231855

**Published:** 2025-11-25

**Authors:** Konstantin Dergilev, Alexander Zubko, Irina Beloglazova, Zoya Tsokolaeva, Ekaterina Azimova, Aleria Dolgodvorova, Irina Iarushkina, Alexander Andreev, Andrey Shiryaev, Pavel Docshin, Anna Malashicheva, Yelena Parfyonova

**Affiliations:** 1 Federal State Budgetary Institution National Medical Research Center of Cardiology Named After Academician E.I. Chazov Ministry of Health of the Russian Federation, 121552 Moscow, Russiakaterinaigma102@gmail.com (E.A.);; 2 A.N. Bakulev National Medical Research Center for Cardiovascular Surgery of the Russian Ministry of Health, 121552 Moscow, Russia; 3 Federal Research and Clinical Center of Intensive Care Medicine and Rehabilitology, Moscow Region, Solnechnogorsk District, v. Lytkino, 141534 Moscow, Russia; 4 Institute of Cytology of the Russian Academy of Science, 194064 St. Petersburg, Russia; pdocshin@icloud.com (P.D.);

**Keywords:** mesothelial cells, epicardium, pericardial fluid, angiogenesis

## Abstract

**Highlights:**

**What are the main findings?**

- Pericardial fluid-derived mesothelial cells (MCs) can be easily obtained from human pericardial fluid and exhibit a characteristic epithelial-like morphology and phenotype, alongside robust proliferation in vitro.
- Pericardial fluid-derived MCs can undergo epithelial-mesenchymal transition (EMT) upon stimulation with TGF-β1, while enhancing the secretion of VEGF, G-CSF, GM-CSF and MCP-3, as well as their proangiogenic properties.

**What are the implications of the main findings?**

- These findings establish a foundation for further investigation into the role of the mesothelium in regulating angiogenesis and cardiac repair mechanisms.

**Abstract:**

Modern therapies aimed at stimulating heart vascularization are critical for regenerating damaged heart tissue and treating ischemic heart disease. Approaches based on developmental biology concepts, particularly those involving the use of cells to coordinate vascular network formation, are of great interest. In this context, epicardial mesothelial cells (MCs) have emerged as a key regulator of blood and lymphatic vessel development during cardiogenesis. However, therapeutic targeting of MCs remains challenging because of anatomical constraints and the difficulties related to isolation of viable cell cultures for research. In this study, we demonstrate for the first time that the pericardial fluid contains cell layers, being an easily accessible source of cardiac MCs. These cells exhibit a characteristic epithelial-like morphology and robust in vitro proliferation, and an ability to undergo epicardial-to-mesenchymal transition in response to TGFβ1. They secrete a broad range of proangiogenic and proinflammatory factors and exert a potent effect on endothelial cells, stimulating proangiogenic behavior and promoting vascular structure formation on Matrigel^TM^. Treating MCs with TGF-β1 enhances the secretion of VEGF, G-CSF, GM-CSF and MCP-3, thereby boosting their proangiogenic properties. Therefore, pericardial fluid is an easily accessible source of MCs for studying their regulatory mechanisms, for being applied in tissue engineering, and for developing approaches to improve heart vascularization.

## 1. Introduction

Coronary artery disease and chronic heart failure remain the leading causes of death worldwide [1,2]. The limited ability of an adult heart to self-repair after ischemic injury, coupled with subsequent scar formation, contributes to the poor prognosis in these patients, particularly in cases of extensive necrosis leading to severe left ventricular dysfunction [3]. Over the past few decades, researchers have sought to stimulate myocardial recovery by targeting various stages of the reparative process, primarily through angiogenesis stimulation [4,5]. Enhancing angiogenesis after myocardial infarction (MI) aims to improve microcirculation by fostering new capillary and collateral arterial vessel formation. Not only does this process protect the myocardium in the acute phase post-MI, but it also mitigates left ventricular remodeling and progression to heart failure at later stages [6,7]. Consequently, the angiogenesis mechanisms and the roles of the key cardiac cells are the focus of active investigation. A coordinated interplay between these factors may regulate neovascularization, restore microvascular perfusion in the heart that has undergone remodeling, and ultimately promote functional recovery.

In this regard, approaches involving the reactivation of evolutionarily conserved, coordinated processes that had initially contributed to the formation and development of heart vessels are of considerable interest [8]. It is particularly noteworthy that the cardiac mesothelium, a continuous layer on the surface of the mature heart (epicardium) that interacts with microvessels located beneath it and surrounds the large coronary arteries, has been identified as a key regulator of vascular network development in the embryonic heart [3,9]. During embryogenesis, the MCs of the epicardium originate from the proepicardium [10], the cells of which migrate to the surface of the developing heart via currents of pericardial fluid [11,12,13]. These cells progressively expand until fully covering the cardiac chambers and activating the EMT program. Through EMT, the MCs differentiates into coronary vessel smooth muscle cells, perivascular mesenchymal cells, and, according to some evidence, coronary endothelial cells [14,15,16,17,18,19,20,21]. The formation of the subepicardial space is also facilitated by the epicardium, thus providing a platform for the assembly of future coronary vasculature [17]. Additionally, MCs of the epicardium secrete factors such as chemokine (C-X-C motif) ligand 12, FGFs, Elabela, VEGFs, PDGFs, angiopoietins, and Thymosin β4 which support coronary vessel morphogenesis [22,23,24,25,26,27,28].

Thus, during early development, the pericardial cavity provides a surface for epicardial MCs, while the mature epicardium forms a highly interactive system. Through coordinated signaling via Notch [29], Sonic hedgehog [30], retinoic acid [31], Wnt/β-catenin [32], PDGF [33], and TGF pathways [34], it regulates heart vessel assembly.

In adults, epicardial MCs remain quiescent but act as a signaling hub for cardiac vascular cell regulation [35]. This role may be mediated by pericardial fluid, which fills the mesothelial-lined pericardial cavity [36]. Initially believed to function merely as a lubricant for cardiac movement, the pericardial fluid is now known to exert both physiological and pathological effects via paracrine signaling [37]. It contains various types of cells and biologically active substances [38,39,40,41], including cardiac hormones, growth factors, prostaglandins, cytokines, and vesicles/exosomes that modulate the cardiac microenvironment, influencing contractility, vascular tone, and cardiac output [38,42,43,44,45]. Numerous studies have characterized the cellular composition of pericardial fluid, detailing both free-floating [38,41,46] and clustered MCs [47]; however, reliable protocols for isolating, cultivating, and functionally evaluating these cells remain scarce.

This study aimed to characterize the MCs derived from free-floating aggregates in human pericardial fluid and to investigate their angiogenic regulatory properties.

## 2. Materials and Methods

### 2.1. Acquisition of Pericardial Fluid Samples

**Figure 1:**
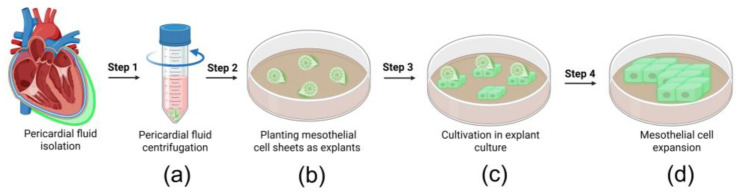
Schematic representation of the procedure for isolating MCs from pericardial fluid samples (Created in BioRender. Derg, K. (2025) https://BioRender.com/qu3c383, accessed on 29 July 2025). (**a**) Isolation of MC sheets by low-speed centrifugation; (**b**) Seeding of mesothelial explants (**c**) Explant culture establishment; (**d**) MCs expansion in culture.

The study enrolled patients from the cardiology center who had undergone coronary artery bypass grafting. Written informed consent was obtained from all participants prior to inclusion. The experiments were approved by the Ethics Committee of the institution (Protocol No. 271 dated 27 September 2021) with a subsequent protocol addendum (Protocol No. 290 dated 29 May 2023). The entire volume of pericardial fluid was obtained from all the patients before the initiation of extracorporeal circulation; it was ensured that it contained no medications. Pericardial fluid samples from patients with a history of diabetes mellitus, inflammatory or rheumatic diseases, patients with active infectious endocarditis, as well as patients who had undergone emergency surgery or repeat surgery were excluded from the study. The characteristics of the patient sample are presented in Supplementary Table S1. Pericardial fluid (20 mL) was collected using a catheter to minimize blood contamination of the samples. After collection, the pericardial fluid samples were transported on ice to the laboratory and immediately used for the mesothelial layer isolation procedure (Figure 1). The processing included 2 min centrifugation at 50× *g*, washing the pellet in PBS, and subsequent three stages of centrifugation (50× *g* for 1 min). The resulting pellet (mesothelial cell sheets) was resuspended in IMDM medium (Servicebio, Wuhan, China) containing 5% FBS (heat inactivated; ATCC), 4 mM L-glutamine, 100 U/mL penicillin-streptomycin (Gibco^®^, Grand Island, NY, USA), with the addition of the inhibitor (10 μM SB 431542 (Miltenyi Biotec, Bergisch Gladbach, Germany)) were seeded as explant cultures on dishes coated with 1% gelatin. Forty-eight hours after planting, the dishes were washed with culture medium to remove non-adherent explants and cultured for 10 days. Every 3 days, 50% of the medium was replaced with fresh medium. Cells were seeded as explant cultures on 1% gelatin-coated dishes. After 48 h, non-adherent explants were removed by medium washing. Cultures were maintained for 10 days with 50% medium replacement every 3 days. Twenty-four hours prior to the experiments, cells were transitioned to basal medium (IMDM with 5% FBS, 4 mM L-glutamine, and 100 U/mL penicillin-streptomycin) after inhibitor washout.

### 2.2. Characterization of Pericardial Fluid-Derived Cell Sheets

Following isolation via low-speed centrifugation, cell sheets derived from pericardial fluid were fixed in formalin and washed with PBS. The layers were then incubated for one hour with the following primary antibodies: Wt1 (Abcam, Cambridge, UK), E-cadherin (Abcam, Cambridge, UK), ZO1 (Abcam, Cambridge, UK), and Notch1 (Sigma, St. Louis, MA, USA). After washing, the samples were incubated for 40 min with the appropriate secondary antibodies (secondary anti-rabbit IgG-Alexa Fluor^®^ 488 or secondary anti-rabbit IgG-Alexa Fluor^®^ 594; used at 1:2000 dilution) (Invitrogen Life Technologies, Carlsbad, CA, USA) at 37 °C. Cell nuclei were counterstained with DAPI. Imaging of the cell sheets was performed using a Leica Stellaris 5 confocal microscope (Leica, Wetzlar, Germany).

### 2.3. Flow Cytometry Analysis

Human mesothelial cells were analyzed for E-cadherin, calretinin, CD34 and CD45 expression via flow cytometry. Briefly, cells were washed with ice-cold PBS and resuspended in PBS with 1% BSA. They were then stained with unlabeled primary antibodies anti-E-cadherin (Abcam, Cambridge, UK), anti-calretinin (ABclonal Biotechnology Co., Ltd., Wuhan, China), anti-CD34 (Santa Cruz Biotechnology, Dallas, USA), anti-CD45 (Abcam, Cambridge, UK), or an IgG isotype control (Abcam, Cambridge, UK) for 30 min at 4 °C, followed by a secondary anti-rabbit Alexa Fluor 647 antibody. After a final wash, cells were fixed in 1% paraformaldehyde. Data was acquired on a BD FACS Aria III (Becton Dickinson, Franklin Lakes, NJ, USA) instrument and analyzed with floreada.io software (WASM Version: SIMD (accessed on 20 July 2025)).

### 2.4. Mesothelial Cell Proliferation Assay

The assay was performed in 96-well plates with cells plated in triplicate at a density of 1000 cells per well. Each plate also contained a serial cell dilution to generate a standard curve. The plates were then assayed every 24 h by adding PrestoBlue™ Cell Viability Reagent (Invitrogen Life Technologies, Carlsbad, CA, USA); fluorescence was measured after a 2 h incubation by Victor3 plate reader (Perkin Elmer, Waltham, MA, USA) at a wavelength of 620 nm. Cell numbers were derived from the standard curve to track proliferation. Each graph represents the average number of cells collected from the pericardial fluid of five patients at the specified time points (24, 48, and 72 h). The population doubling time was calculated using the algorithm available at https://doubling-time.com/compute_more.php (accessed on 20 July 2025).

### 2.5. RNA Isolation, Reverse Transcription and Real-Time Quantitative PCR

RNA was extracted from pericardial fluid-derived MCs (*n* = 4) for gene expression analysis. Cells were lysed in RLT buffer and homogenized, followed by RNA isolation using the RNeasy Mini Kit (QIAGEN, Germantown, MD, USA) with on-column DNase I digestion. After quantification on a NanoDrop 2000 (ThermoFisher Scientific, Waltham, MA, USA), 1 µg of total RNA was reverse-transcribed into cDNA using a RevertAid First Strand cDNA Synthesis Kit (ThermoFisher Scientific, Waltham, MA, USA). Gene expression was quantified by real-time PCR on a StepOnePlus system (ThermoFisher Scientific, Waltham, MA, USA) using SYBR Green chemistry (Evrogen, Moscow, Russia). Gene expression levels were normalized to GAPDH and analyzed via the 2−ΔΔCt method. The corresponding primer sequences are listed in Table 1.

### 2.6. Protein Extraction and Western Blotting

Using ice-cold RIPA buffer (50 mM Tris–HCl pH 7.4, 150 mM NaCl, 1 mM EDTA, 1% Triton X-100, 0.5% sodium deoxycholate, 0.1% SDS) containing protease and phosphatase inhibitors, we prepared protein lysates from pericardial fluid-derived MCs (*n* = 4). After scraping and centrifugation, the supernatants were mixed with 6× sample buffer and denatured. Proteins were separated by SDS-PAGE on 6% gels and transferred to PVDF membranes (Merck KGaA, Darmstadt, Germany). Membranes were blocked with 5% non-fat milk in TBST and subsequently incubated overnight at 4 °C with primary antibodies anti-Tubulin (Cell Signaling, Boston, MA, USA), anti-Notch1 (Cell Signaling, Boston, MA, USA), anti-E-cadherin (Abcam, Cambridge, UK) and anti-Jagged1 (ABclonal Biotechnology Co., Ltd., China). Following incubation with HRP-conjugated II antibodies, protein bands were visualized using a FusionFX gel-documentation system (Vilber Lourmat, Collégien, France). Protein expression levels were normalized to Tubulin and quantified using ImageJ software (v1.54d, NIH).

### 2.7. Condition Medium Harvesting

Conditioned media were collected from both control pericardial fluid-derived MCs and TGF-β1 (2 ng/mL)-treated MCs. After a DPBS wash, cells were cultured for 24 h in serum-free IMDM medium containing penicillin-streptomycin. The CM was then centrifuged, and the clarified supernatants were aliquoted and stored at −70 °C.

### 2.8. Magpix Analysis

Cytokine/Chemokine levels in conditioned media (control and TGF-treated human MCs derived from 3 different donors) were quantified using the MILLIPLEX MAP 41-Plex Human Cytokine/Chemokine Magnetic Bead Panel (Merck KGaA, Darmstadt, Germany) per the manufacturer’s protocol, as previously detailed [48]. Briefly, conditioned media samples, serially diluted standards, and controls were loaded in duplicate into a 96-well plate. After adding the antibody-conjugated magnetic beads, the plate was incubated overnight at 4 °C. Following a wash step, a biotinylated detection antibody was applied, and the signal was amplified with streptavidin-phycoerythrin. Fluorescence was measured on a MAGPIX^®^ System (Merck KGaA, Darmstadt, German), and cytokine concentrations were determined from the standard curve using xPONENT software (v4.3.229.0).

### 2.9. In Vitro Angiogenesis Assay

A tube formation assay using HUVECs was performed to evaluate angiogenic potential, as previously detailed [49,50]. HUVECs (5 × 10^4^ cells/cm^2^) were plated on a Matrigel™ (Corning, Corning, NY, USA) matrix and incubated for 18 h with IMDM (unconditioned) medium (Gibco^®^, Grand Island, NY, USA), EGM-2 media (Lonza, Basel, Switzerland), conditioned medium from untreated MCs, and conditioned medium from TGF-β1-treated MCs. Post-assay, cells were fixed with 4% formaldehyde and visualized using an Image Exfluorer AI microscope (Life Cell Instrument, Seoul, Republic of Korea) with a life-maintaining module (37 °C, 5% CO_2_). Tube networks were imaged live, and key parameters (total tube length, number of segments) were quantified with ImageJ software (v1.54d, NIH).

### 2.10. Matrigel^TM^ Plug Assay

To evaluate the angiogenic potential of mesothelial cells in vivo, a Matrigel™ plug assay was conducted in 8-week-old BALB/c nude mice. The mice were randomly assigned to three experimental groups: one received injections of growth factor-reduced Matrigel™ mixed with unsupplemented IMDM (control), second group received Matrigel™ mixed with human MCs (4 × 10^5^ cells resuspended in IMDM), while the other received Matrigel™ mixed with TGFb1-treated MCs (4 × 10^5^ cells resuspended in IMDM). The mixtures were injected subcutaneously into the bilateral flanks (groin area) of each mouse. After 14 days, the Matrigel plugs were harvested and processed for analysis. To quantify vascularization, cryosections of the plugs were immunostained for the endothelial marker CD31. Briefly, sections were fixed in acetone, blocked with 10% normal donkey serum, and incubated with a primary rat anti-mouse CD31 antibody (BD Pharmingen, BD Bioscience, San Jose, CA, USA). This was followed by incubation with an Alexa Fluor 488-conjugated II antibody (Life Technologies, ThermoFisher Scientific, Waltham, MA, USA) and counterstaining of nuclei with DAPI. The area of the CD31-positive area relative to the standard field of view of the Matrigel™ plug was quantified to assess vascularization using the General Analysis 3 module in NIS-Elements software (v5.42.01).

Matrigel^TM^ vascularization was additionally evaluated using CD31 immunohistochemistry on cryosections. Staining was performed using ImmPRESS polymer reagents with a red phosphatase substrate (Vector Laboratories, Newark, CA, USA), according to the manufacturer’s protocol. CD31 primary antibody was from Becton Dickinson (Franklin Lakes, NJ, USA). Nuclei are counterstained with hematoxylin (blue). Images were acquired using a Leica Aperio CS2 system (Leica, Wetzlar, Germany).

### 2.11. Statistical Analysis

Values represent the mean ± standard deviation of a minimum of three independent experiments. Normality was assessed using the Shapiro–Wilk test. Differences between samples with normal distribution were determined by one-way or two-way (secretome analysis) ANOVA, while Mann–Whitney test was used to compare non-normal data in GraphPad Prism (v8). A *p*-value below 0.05 was deemed statistically significant.

## 3. Results

### 3.1. Isolation of Epicardial MCs Derived from Pericardial Fluid Samples

All the patients undergoing coronary artery bypass grafting and participating in this study underwent a minimally invasive pericardial fluid collection procedure (Figure 1).

**Figure 2:**
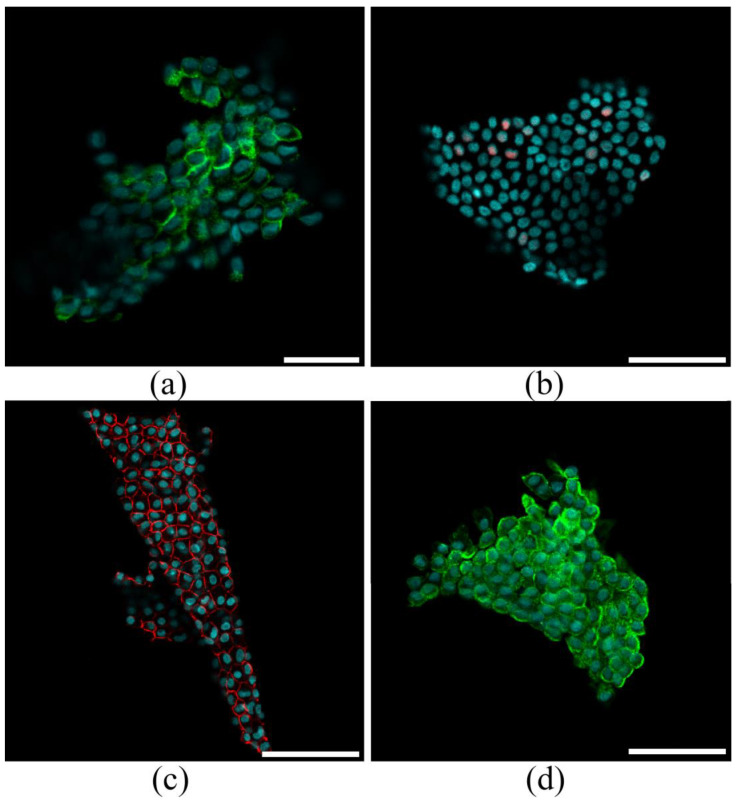
Characteristics of MC layers derived from pericardial fluid samples. (**a**–**d**) representative images of MC layers stained against Notch 1 ((**a**); green), Wt1 ((**b**); red), ZO-1 ((**c**); red) and E-cadherin ((**d**); green). Cell nuclei were counterstained with DAPI (blue). Scale bar = 50 µm.

Following sternotomy, the fluid was aspirated using a catheter, minimizing blood contamination and ensuring high sample integrity. The procedure was straightforward and complication-free, demonstrating its feasibility and safety. All pericardial fluid samples contained MC sheets, which were pelleted via low-speed centrifugation (Figure 1, Step 1). The MC sheets consisted of cells that expressed Notch1 (Figure 2a) and interacted through contacts involving E-cadherin and ZO-1 (Figure 2c,d). The low expression of the mesothelial marker WT1 (in less than 15% of cells; Figure 2b) indicated that the layers retained their epithelial-like characteristics.

These cell layers were then plated as explants and cultured to establish epicardial MC lines. Figure 1 summarizes the key steps of our isolation protocol.

### 3.2. Characterization of Pericardial Fluid-Derived MCs

**Figure 3:**
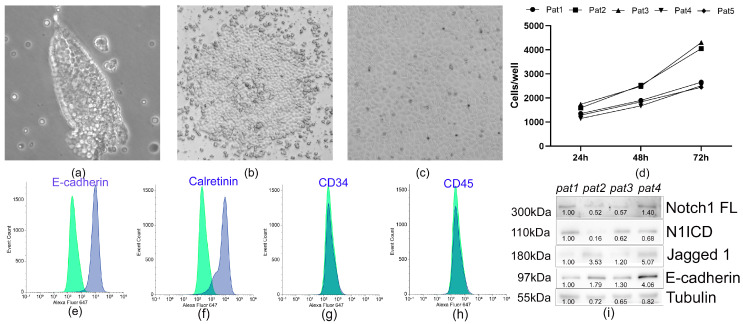
(**a**–**c**) Characterization of pericardial fluid-derived MCs (**a**) Representative images showing MC culture stages: cell layers; (**b**) 2-day explant culture; (**c**) 10-day MC culture; (**d**) Growth kinetics of cardiac MCs; (**e**–**h**) Flow cytometry analysis of immunophenotypic markers. Blue histograms represent cells stained with antibodies against (**e**) E-cadherin, (**f**) Calretinin, (**g**) CD34, and (**h**) CD45; green histograms show IgG isotype controls; (**i**) Immunoblotting data show the expression of Notch1 FL (full length), N1ICD, Jagged1, E-cadherin, and tubulin proteins in MCs obtained from pericardial fluid. All the data were obtained from cells isolated from four patients (pat).

MC layers were isolated via low-speed centrifugation and established as explant cultures. Within 48 h post-seeding, cells started migrating from the primary explants to form a primary culture (Figure 1, step 2). By day 4, the population exhibited a mixed morphology of mosaic and elongated cells (Figure 1, step 3; Figure 2b). Confluent cultures had been formed by day 14, with cells displaying the characteristic cobblestone morphology typical of MCs (Figure 1, step 4; Figure 3c). The cells exhibited moderate proliferation rates, with an average doubling time of 45 ± 9 h (*n* = 5) (Figure 3d). An immunophenotypic analysis revealed strong expression of E-cadherin (92.4 ± 7.6%; *n* = 5) and Calretinin (86.9 ± 8.9%; *n* = 5), while being negative for CD45 and CD34 (Figure 3e–h). Immunoblotting confirmed expression of Notch1 receptor and its ligand Jagged1 (Figure 3i), which are known to regulate cell fate decisions, modulate signaling pathways, and mediate cellular crosstalk.

### 3.3. Pericardial Fluid-Derived MCs Exhibit an Ability to Undergo EMT and Modulate Their Secretion

Studies focusing on the effects of TGF-β on MC function and tissue homeostasis have become an important area of research due to its pivotal role in tissue repair, fibrosis, and immune modulation of serous surfaces [51,52].

**Figure 4:**
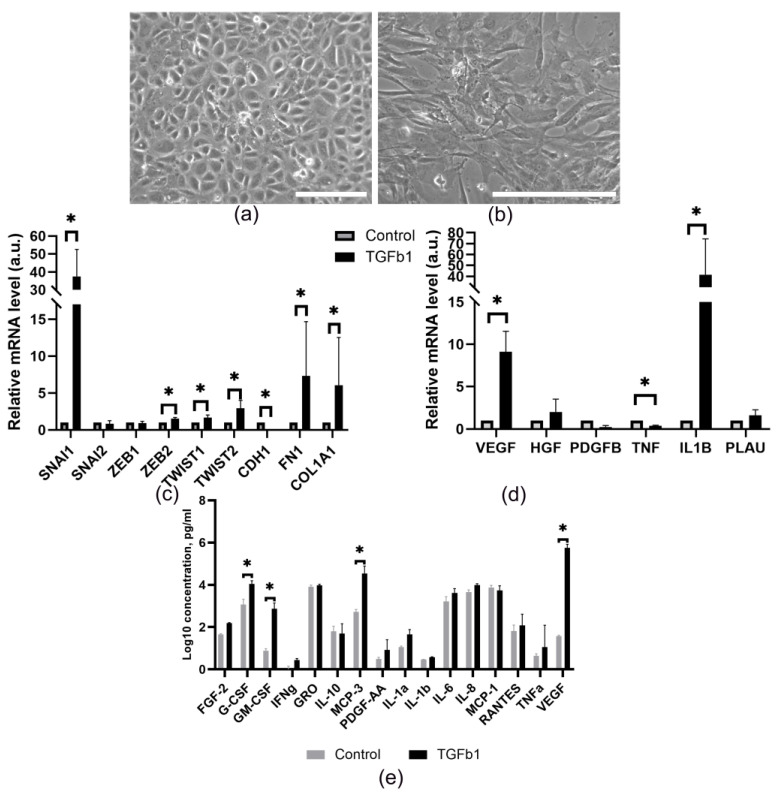
TGF-β1 induces the EMT and alters functional properties of MCs. The representative morphology of cells (**a**) before and (**b**) after TGF-β1 treatment (2 ng/mL, 48 h). Scale bar represents 100 μm; (**c**) qRT-PCR analysis of EMT-related gene expression in TGF-β1-stimulated cells (Day 2 post-treatment). Data show the fold change in mRNA levels versus untreated controls (* *p* < 0.05, *n* = 3); (**d**) qRT-PCR analysis of growth factor gene expression following TGF-β1 stimulation (Day 2). Data are presented as the fold change versus control (* *p* < 0.05, *n* = 3); (**e**) The cytokine/chemokine secretion profile in conditioned medium from untreated and TGF-β1-treated (2 ng/mL) MCs. Values represent mean concentrations from triplicate experiments.

Recent studies have shown that TGF-β levels may be elevated in the pericardial fluid [53,54,55]. In this context, we investigated how TGF-β affects the properties of MCs. We found that TGF-β causes prominent changes in the morphology of MCs (Figure 4a,b): they lose their cobblestone morphology and acquire a spindle-shaped appearance. RT-PCR analysis (Figure 4c) revealed an increased expression of EMT regulatory genes (SNAI1, ZEB2, TWIST1, TWIST2) and mesenchymal markers (FN1, COL1A1), along with a decrease in CDH1 expression. These data are indicative of induction of EMT characteristics in MC cultures, which enhances the cells’ migratory and invasive abilities and promotes their biodistribution. Consistent with previous findings, EMT activation also induces changes in the cells’ secretory profile [56,57]. Neovascularization, which depends on various types of cells and inflammation, appears to be caused by massive, pleiotropic cellular and molecular reactions that induce changes in the expression patterns of pro-inflammatory and proangiogenic genes. We found that TGF-β1-induced MC transition elevated mRNA expression of the key angiogenic factors, VEGF and IL1B. In contrast, TNF expression remained at control levels. The expression of other vascularization mediators (PLAU, HGF, PDGFB) was also unchanged, indicating that TGF-β1 triggers a highly specialized response in MCs.

Furthermore, we analyzed the protein profile in the culture medium (Figure 4e) using the MILLIPLEX MAP 41 Human Cytokine/Chemokine Magnetic Bead Panel and found that these cells secrete a wide range of proangiogenic and proinflammatory factors. Treatment with TGF-β1 enhanced the secretion of: VEGF, G-CSF, GM-CSF and MCP-3, but did not alter the TNFa, IL-1α, RANTES, EGF, IP-10, MIP-1α, IFN-γ, MDC, PDGF-AA, IL-9, IL-4, or MIP-1β levels. Notably, TGF-β1 stimulation increased the VEGF and MCP-3 levels by more than 15,000-fold and 70-fold, respectively, versus the baseline. Hence, not only does TGF-β1 induce EMT, but it also significantly enhances the secretion of proangiogenic/proinflammatory factors by MCs in vitro.

### 3.4. The Proangiogenic Potential of Pericardial Fluid-Derived MCs in Vascular Network Formation

**Figure 5:**
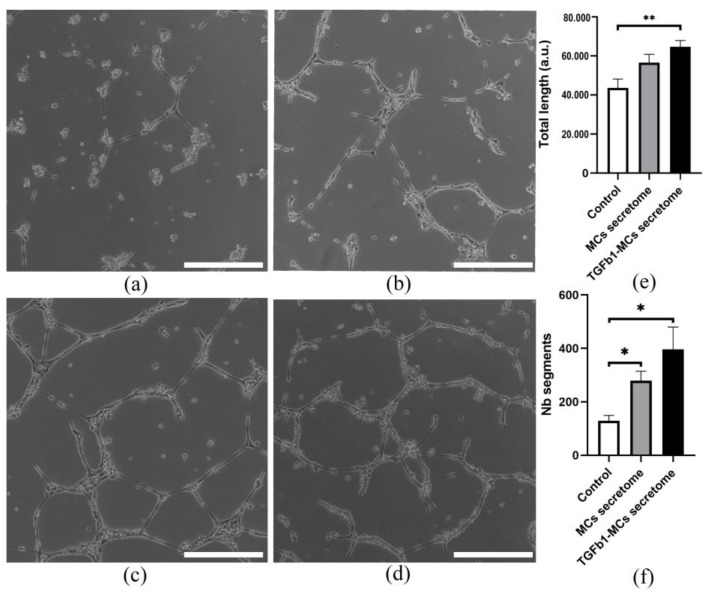
In vitro HUVEC tube formation assay (**a**–**d**) The representative images of the tubular structures formed by HUVECs over 18 h (**a**) in IMDM (unconditioned) medium, (**b**) in control (EGM medium), (**c**) MCs condition medium and (**d**) TGFb1-treated MCs condition medium; Scale bar = 250 µm; (**e**) Graph of the total tube length over 18 h in control and medium conditioned by MCs and TGFb1-treated MCs; (**f**) Graph of the tube segments number over 18 h in control and medium conditioned by MCs and TGFb1-treated MCs; The asterisk (*) indicates a significant difference (* *p* < 0.05, ** *p* < 0.01) between groups (*n* = 3).

Consistent with our hypothesis, mesothelial cells exhibit a significant potential for vascular network restoration through their proangiogenic effects on endothelial cells, mediated by specialized secretome. In order to evaluate this hypothesis, the proangiogenic properties of MCs and TGFb1-treated MCs were assessed in both in vitro and in vivo tests. In vitro, conditioned media collected from MCs and TGFb1-treated MCs were incubated with HUVEC on Matrigel-coated dishes (Figure 5c,d). Despite inducing a trend toward increased vascular network length, the activity of the mesothelial secretome across different patient environments was comparable to that of the EGM control, while the number of tube segments exceeded control values (Figure 5e,f). Although TGFβ1 stimulation of MCs further boosted their proangiogenic activity across all parameters, the effect was not statistically significant versus the unstimulated secretome (Figure 5c,d).

**Figure 6:**
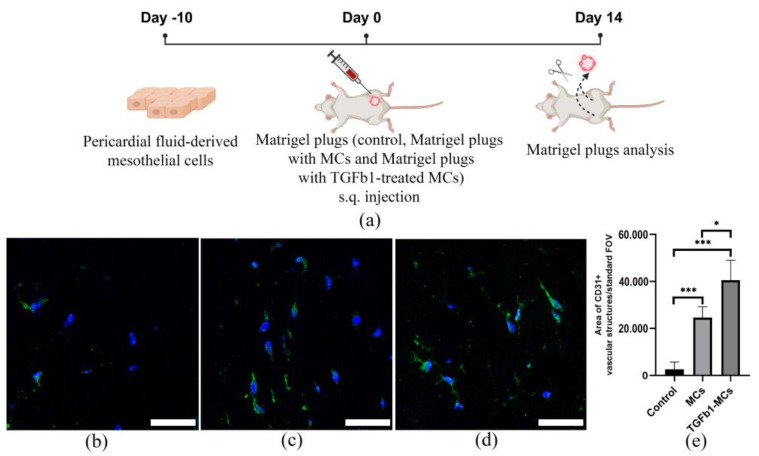
In vivo angiogenesis within Matrigel^TM^ plugs. (**a**) The representative design scheme of the experimental study (https://app.biorender.com accessed on 29 July 2025). Matrigels with IMDM (control), MCs or TGFb1-treated MCs were injected subcutaneously in the groin area. After 14 days, plugs were recovered by dissection. (**b**–**d**) The representative images of (**b**) control Matrigel^TM^ plugs, (**c**) Matrigel^TM^ plugs with MCs and (**d**) Matrigel^TM^ plugs with TGFb1-treated MCs stained with antibodies against endothelial marker CD31 (green). The nuclei were stained with DAPI (blue). (**e**) Quantitative analysis of the area of CD31+ vascular structures in the standard field of view. * *p* < 0.05, *** *p* < 0.005. Scale bar represents 50 μm.

An in vivo Matrigel plug assay was used to assess proangiogenic potential of MCs. MCs and TGFβ1-treated MCs were suspended in Matrigel and subcutaneously transplanted into immunodeficient mice (Figure 6a). After fourteen days, the area of CD31+ vessel-like structures in plugs containing either MCs or TGFβ1-treated MCs was more than 9 and 15 times higher, respectively, than in the cell-free Matrigel control (Figure 6b–e; Supplementary Figure S1). Furthermore, the proangiogenic activity of TGFβ1-treated MCs was significantly greater than that of untreated MCs.

## 4. Discussion

Our understanding of epicardial MCs has evolved significantly, revealing their central role in maintaining cardiac homeostasis. These cells form an intricate regulatory network coordinating myocardial cell interactions and mediating intercellular signaling [35]. Through multiple mechanisms, including direct cell contact, paracrine signaling, and pericardial fluid modulation, MCs orchestrate cardiac adaptation to regional stimuli [53,58,59]. The pericardial fluid serves as a critical medium rich in bioactive molecules such as microvesicles/exosomes, hormones, neurotransmitters, cytokines, and growth factors [60,61].

In this study, we demonstrated for the first time that the pericardial cavity contains cell sheets that can be an easily accessible source of MCs. Second, the obtained MCs have a characteristic epithelial-like morphology and phenotype. In addition, these cells proliferate well in vitro and are capable of entering the EMT under the action of TGFb1. Third, MCs in epithelial and mesenchymal states secrete a rich set of proangiogenic/proinflammatory biologically active factors. Finally, TGF-β1-treated MCs exhibit a more potent proangiogenic effect, driving more extensive growth of vessel-like structures in Matrigel^TM^ plugs than their native counterparts.

Similar to the adult heart, the pericardial fluid plays a crucial role in the redistribution of MCs during embryogenesis. This function ensures the uniform distribution of these cells on the surface of the developing heart, contributing to its proper formation [11,12]. During this period, the mesothelium exhibits epithelial-like characteristics, similar to those of cells derived from the adult pericardial cavity. These include apical-basal polarity, tight junction-mediated intercellular interactions, basement membrane adhesion, surface microvilli, and the capacity to undergo EMT. EMT generates progenitor cells (EPDCs) and enhances secretory activity, both being essential for heart maturation [60].

Moreover, MCs differentiate into fibroblasts, smooth muscle cells, and pericytes, which are critical for coronary vessel formation [62,63]. Despite the complexity of the signaling pathways regulating vasculogenesis and the contributions of diverse cell sources such as the sinus venosus and endocardium [19,64], a distinct subpopulation of epicardial derivatives marked by Scleraxis and Semaphorin3D expression forms the coronary endothelium [20]. Their specification is likely to be regulated by changes in the ECM synthesized by the epicardium and its derivatives, as well as by intracellular and pericardial fluid-derived signaling molecules [65]. The epicardium also governs coronary lymphatic development. It secretes VEGF C, a lymphangiogenic cytokine, and conditional removal of epicardial VEGF C abrogates lymphatic network formation [66,67].

During early development, the epicardial mesothelium acts as a source of EPDCs, extracellular matrix proteins, and secretory signals essential for vascular network formation. Additionally, it generates the subepicardial space, creating a specialized microenvironment where coronary vessel precursors assemble. Hence, coronary network development is intrinsically linked to—and dependent on—proper epicardial formation [68]. In adults, the mesothelium is typically a slowly renewing, quiescent cell type. However, it retains the capacity to reactivate embryonic repair programs upon stimulation, recapitulating developmental processes. This plasticity suggests that the epicardium could be a therapeutic target for vascular regeneration and cardiac repair [69,70]. Notably, we observed that entire layers of MCs float within the pericardial fluid. This phenomenon likely reflects the unique reparative mechanism for the epicardium/pericardium, wherein detached cell layers from distant regions adhere to restore the lining, a process analogous to repair in other serous cavities [71,72]. Detachment of these layers may be mediated by the Notch signaling pathway [73]. MCs express Notch1 receptor and Jagged1 ligand, key components of this evolutionarily conserved pathway, which regulates angiogenesis, proliferation, differentiation, and secretory activity [74,75]. Our recent work demonstrates that Notch-dependent intercellular signaling modulates type IV collagen expression—a critical basement membrane component, potentially influencing membrane stability and selective mesothelial layer detachment [76].

Signal transduction is another potential function of the cell layers in the pericardial cavity. Numerous ligands and receptors participate in NOTCH signaling, exhibiting temporally and spatially restricted expression across diverse tissues. We hypothesize that MC migration into the pericardial cavity and their ligand-receptor-mediated interactions with epicardial cells may determine cell fate and maintain physiological homeostasis. These interactions could occur via classical and non-classical mechanisms, including crosstalk with the NICD-NF-κB, NICD-mTOR, NICD-PTEN, NICD-AKT, NICD-Wnt, NICD-Hippo, and NICD-TGF-β pathways [75]. Critically, epicardium-derived MCs retain their epithelial-like morphology and phenotype in vitro. They exhibit robust proliferation and can undergo epithelial–mesenchymal transition (EMT) in response to TGF-β1, a key mediator of this process. TGF-β1 upregulates the transcription factor SNAI1, which strongly suppresses epithelial markers (e.g., E-cadherin and keratins) [50,76]. EMT activation generates a fibroblast-like, motile phenotype, resembling that of embryonic cells. These properties may reflect an evolutionarily conserved mechanism for postnatal epicardial repair. Notably, MC layers or individual cells adhering to injured cardiac surfaces could initiate partial EMT, enabling cell redistribution and amplification to repair epicardial defects. Supporting this, two out of seventeen MC samples isolated from the pericardial fluid in vitro predominantly adopted a mesenchymal morphology (unpublished data), likely due to pericardial fluid-derived factors [53,54,55]. These factors may reprogram cell properties, enhancing their integration into the epicardial niche.

A notable finding of this study was that MCs exhibited a high secretory activity. The formation of a gradient of proinflammatory cytokines in the microenvironment can directly stimulate vessel formation by interacting with target endothelial cells or indirectly by stimulating leukocytes and/or endothelial cells to produce proangiogenic mediators. Along with it, secretion of the key proangiogenic factors (Figure 3) identified as drivers of proangiogenic behavior of endothelial cells was confirmed in our work using a combination of in vitro and in vivo methods (Figure 4 and Figure 5). These data align with findings reported by other research groups [77], suggesting that MCs from diverse sources may contribute to tissue repair processes by secreting a broad range of cytokines, growth factors, and extracellular matrix molecules, including collagen types I, III, and IV, elastin, fibronectin, and laminin [78,79,80].

It is well known that MCs secrete TGFb, PDGFs, FGF family members (FGF1, 2, 4, 9, 16, and 20), HGF, KGF, and members of the endothelial growth factor family (EGF, heparin-binding EGF, and VEGF), which promote endothelial growth [81,82,83]. Furthermore, these cells have been shown to stimulate autocrine and paracrine proliferation, differentiation, and migration of various tissue-specific cell types [60]. This secretory activity can occur via spontaneous diffusion (for molecules with a molecular weight ≤40 kDa) through the epicardium or through transport via specialized structures called stomata associated with subepicardial vessels [84,85]. We hypothesize that pericardial MC secretion contributes to composition of the pericardial fluid and may regulate the state of cardiac vascular cells. In this study, we demonstrated that epithelial- and mesenchymal-state MCs secrete a rich array of proangiogenic and proinflammatory bioactive factors, stimulating vascular structure growth both in vitro and in vivo. This effect may be mediated by proangiogenic factors such as FGFs [23,24], VEGFs [86], GM-CSF, G-CSF [87], and MCP-3 present in the MCs secretome [88]. An important finding of our study is that TGF-β1 stimulation, which is significantly elevated in pericarditis and ischemic heart disease [53,54], alters the morphology of MCs, the composition of their secretome and enhances their proangiogenic properties. These changes may reflect the involvement of these cells in both the regulation of angiogenesis and pericardial remodeling associated with the development of pericardial adhesions. Specifically, the loss of epithelial-like characteristics prompts mesothelial cells to form membrane bridges, creating the foundation for adhesions [84,85]. Concurrently, TGFβ1-induced secretion of VEGF and other proangiogenic factors contributes to adhesion vascularization [48], thereby propagating the condition.

Furthermore, proinflammatory factors secreted by MCs may modulate composition of the pericardial fluid and potentially influence the phenotypes of inflammatory cells, such as macrophage activation and the formation of neutrophil extracellular traps via NF-κB, NLRP3 inflammasome, and chemokine signaling [89,90]. Further studies evaluating individual factors and identifying specialized microRNAs could provide a comprehensive understanding of the immune modulation mechanisms [89,91]. Another promising research direction involves identifying mesothelial secretory signatures that may act as diagnostic markers for postoperative inflammatory complications, enabling a more nuanced understanding of clinical outcomes and therapeutic responses.

The current study has several limitations. The main limitation is that pericardial fluid was sourced from cardiac surgery patients, as ethical constraints prevented its collection from healthy individuals. To approximate a “normal” sample, only stable patients undergoing scheduled surgery and showing no signs of active inflammation or pericardial disease were included. A further important limitation is the lack of large-scale analysis on the interaction between the MC secretome and pericardial fluid composition. Additionally, more research is needed to determine how epicardial derivatives, particularly fibroblasts, and other pericardial cell types influence the multicomponent composition of pericardial fluid over the course of ischemic heart disease and after surgery. Such investigations are crucial to understand the regulatory mechanisms governing the properties of pericardial cavity cells, the condition of the coronary vessels, and the cardiac microenvironment as a whole. Additionally, the proangiogenic effect observed in both untreated and TGF-β1-activated MCs highlights the need for a more detailed assessment of their impact on key endothelial cell functions, such as proliferation, migration and barrier permeability, and for testing their effects in a cardiac injury models in vivo. Further single-cell transcriptomic and proteomic analysis of endothelial cells would help distinguish the effects of the mesothelial cell secretome from those of other pericardial cells, greatly advancing our understanding of cardiac vascular regulation. Furthermore, the characterization of mesothelial cell derivatives, particularly fibroblasts and myofibroblasts, following TGF-β1-induced EMT is a critical next step. These studies are crucial for clarifying the role of the mesothelium in regulating post-infarction myocardial revascularization and angiogenesis during pericardial adhesion formation and fibrosis development.

## 5. Conclusions

In conclusion, we propose using pericardial fluid for straightforward and efficient isolation of human MCs. The proangiogenic properties of these cells (in their basal state and after treatment with TGFb1), demonstrated in this study, suggest their potential for future research and as a platform for novel therapies aiming to stimulate angiogenesis. With further in vivo evidence, MCs transplantation following aortocoronary bypass surgery could emerge as a therapeutic strategy to enhance myocardial vascularization, improve graft survival, and reduce postoperative adhesion-related complications.

Additional research could focus on investigating the application of cells derived from the pericardial cavity as an alternative to mesothelial-like induced pluripotent stem cells or induced proliferative cells derived from the adult human epicardium to overcome the limitations associated with obtaining large quantities of cells [92,93]. Further validation of transplantation conditions, dosage, and optimal proangiogenic effects will be essential for clinical translation.

## Figures and Tables

**Table 1:** Primers.

Gene Name	Forward	Reversed
*SNAI1*	TCGGAAGCCTAACTACAGCGA	AGATGAGCATTGGCAGCGAG
*SNAI2*	CGAACTGGACACACATACAGTG	CTGAGGATCTCTGGTTGTGGT
*ZEB1*	TTACACCTTTGCATACAGAACCC	TTTACGATTACACCCAGACTGC
*ZEB2*	GCGATGGTCATGCAGTCAG	CAGGTGGCAGGTCATTTTCTT
*TWIST1*	GTCCGCAGTCTTACGAGGAG	GCTTGAGGGTCTGAATCTTGCT
*TWIST2*	TCTGAAACCTGAACAACCTCAG	CTGCTGTCCCTTCTCTCGAC
*CDH1*	ATTTTTCCCTCGACACCCGAT	TCCCAGGCGTAGACCAAGA
*FN1*	GACGCATCACTTGCACTTCT	GCAGGTTTCCTCGATTATCCT
*COL1A1*	CCAAATCTGTCTCCCCAGAA	TCAAAAACGAAGGGGAGATG
*VEGFA*	CAACATCACCATGCAGATTATGC	GCTTTCGTTTTTGCCCCTTTC
*HGF*	AGGGGCACTGTCAATACCATT	CGTGAGGATACTGAGAATCCCAA
*PDGFB*	TCCCGAGGAGCTTTATGAGA	GGGTCATGTTCAGGTCCAAC
*TNFA*	ATGAGCACTGAAAGCATGATCC	GAGGGCTGATTAGAGAGAGGTC
*IL1B*	ACAGATGAAGTGCTCCTTCCA	GTCGGAGATTCGTAGCTGGAT
*PLAU*	CTCCTGTGCATGGGTGAA	AACCATGGGCCTCACAAAT
*GAPDH*	TGCACCACCAACTGCTTAGC	GGCATGGACTGTGGTCATGAG

## Data Availability

The original contributions presented in this study are included in the article. Further inquiries can be directed to the corresponding author.

## Supplementary Materials

The following supporting information can be downloaded at: https://www.mdpi.com/article/10.3390/cells14231855/s1, Figure S1: Representative images of MatrigelTM plugs cryosections ((a) Control MatrigelTM plug; (b) MatrigelTM Plug containing MCs), immunohistochemically stained for the endothelial marker CD31 (red). Nuclei are counterstained with hematoxylin (blue). Scale bar represents 100 μm; Table S1: Characteristics of the patient sample.

## Abbreviations

The following abbreviations are used in this manuscript:

BMI	Body mass index
BSA	Bovine serum albumin
CM	Condition medium
DAPI	4′,6-diamidino-2-phenylindole
DM	Diabetes mellitus
DPBS	Dulbecco’s Phosphate-Buffered Saline
ECM	Extracellular matrix
EGF	Epidermal Growth Factor
EMT	Epithelial-to-mesenchymal transition
EPDCs	Epicardium-derived cells
ET-1	Endothelin-1
FGF	Fibroblast growth factor
GAPDH	Glyceraldehyde 3-phosphate dehydrogenase
G-CSF	Granulocyte colony-stimulating factor
GM-CSF	Granulocyte-macrophage colony-stimulating factor
GRO-1	Growth-Regulated oncogene 1
HC	Hypercholesterolemia
HGF	Hepatocyte growth factor
HT	Hypertension
HUVEC	Human umbilical vein endothelial cells
IFN-γ	Interferon gamma
IL-1α	Interleukin-1alpha
IL-4	Interleukin-4
IL-6	Interleukin-6
IL-9	Interleukin-9
IP-10	Interferon Gamma-induced Protein 10
IMDM	Iscove’s Modified Dulbecco’s Medium
KGF	Keratinocyte growth factor
MC	Mesothelial cell
MCAD	Multivessel coronary artery disease
MCP-3	Monocyte chemotactic protein-3
MDC	Macrophage-derived chemokine
MIP-1α	Macrophage inflammatory protein 1-alpha
mTOR	Mammalian target of rapamycin
NICD	Notch Intracellular Domain
NF-κB	Nuclear factor kappa-B
Pat	Patient
PBS	Phosphate-Buffered Saline
PDGF	Platelet-derived growth factor
PTEN	Phosphatase and Tensin homolog
RANTES	Regulated on activation, normal T-cell expressed and secreted
TGFb	Transforming growth factor beta
TNF-α	Tumor necrosis factor-α
VEGF	Vascular endothelial growth factor
