# Supplementary figures and images for: Human Pericardial Fluid-Derived Cells Exhibit Mesothelial-like Properties and Exert Proangiogenic Effects on Endothelial Cells
Source: Cells. 2025 Nov 25;14(23):1855. doi: 10.3390/cells14231855 (PMC12691167; doi:10.3390/cells14231855)

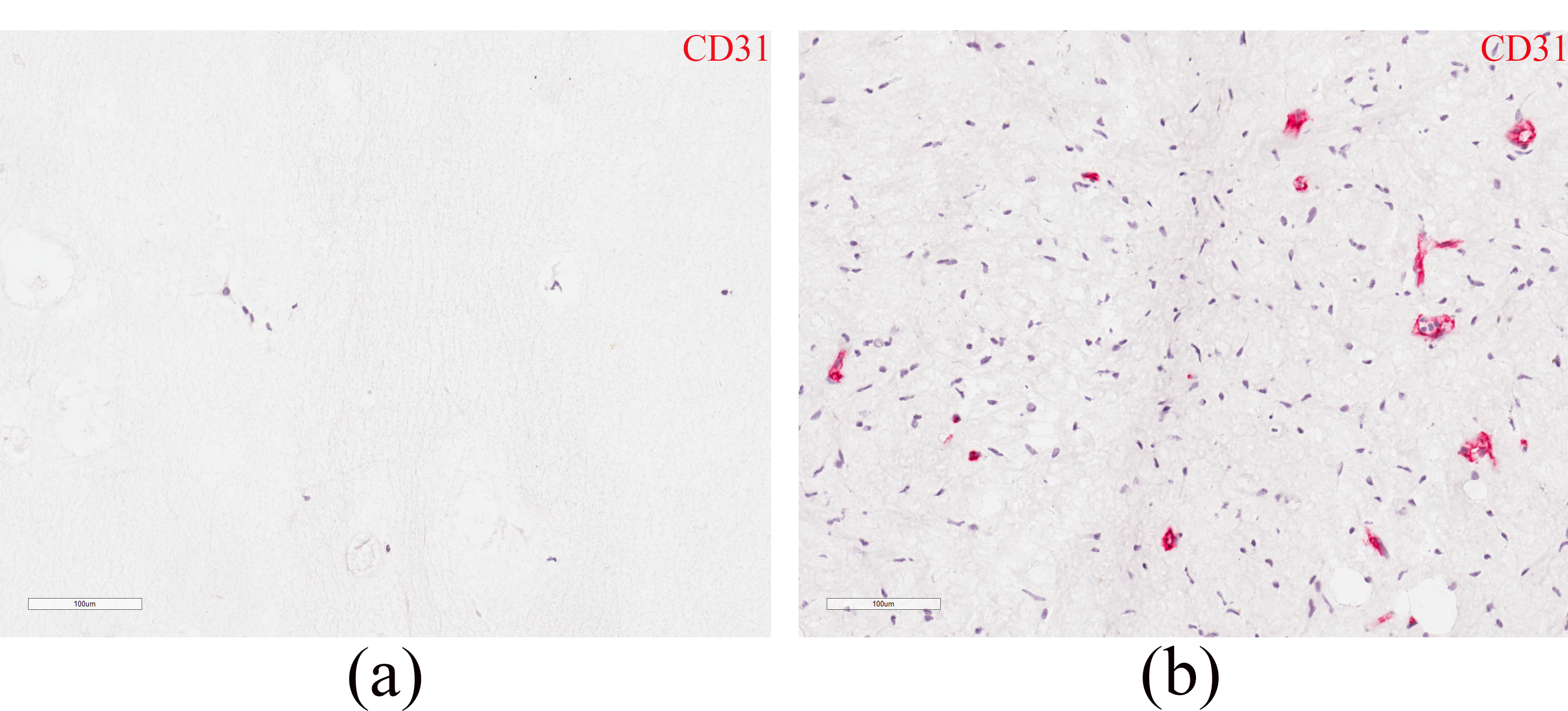

Supplement: Supplementary file 1 [file cells-14-01855-s001.zip › Supplementary figure 1.tif]

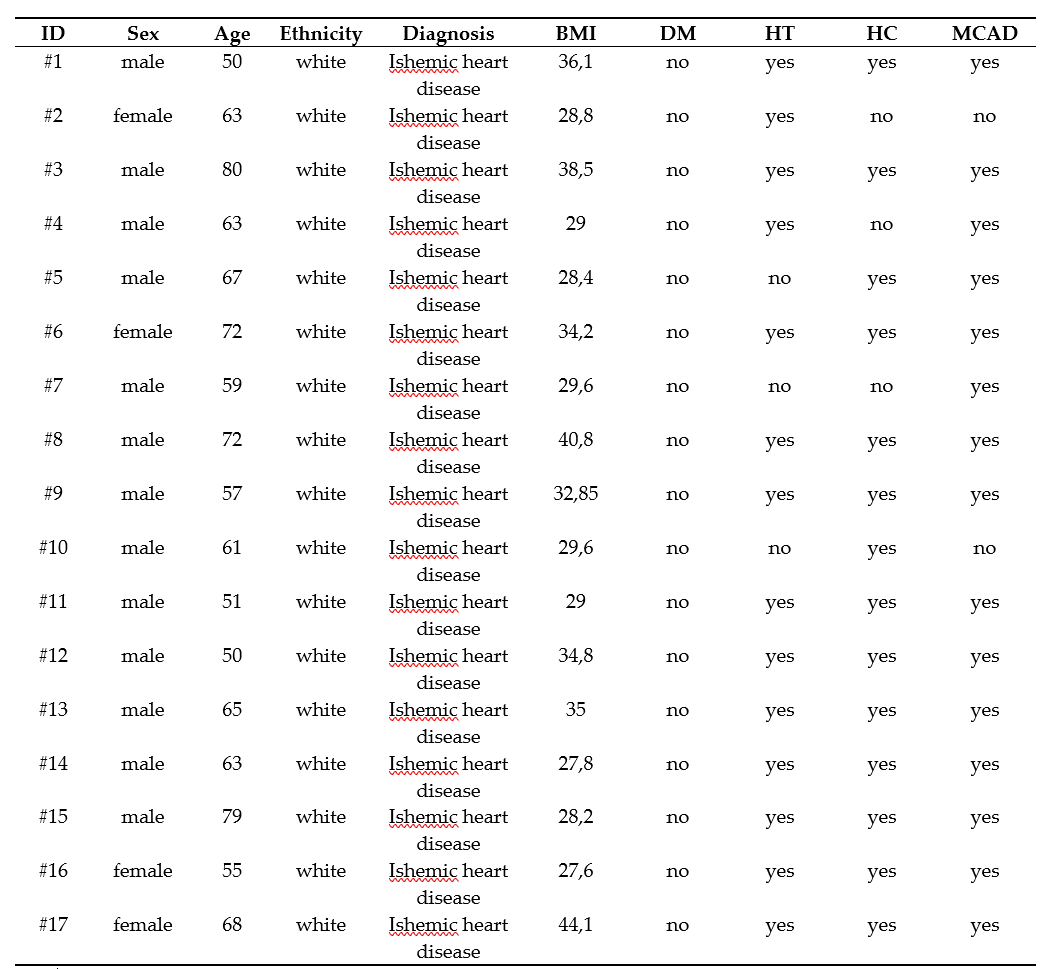

Supplement: Supplementary file 1 [file cells-14-01855-s001.zip › Supplementary table 1.tif]
